# The role of tumor immune microenvironment in chordoma: promising immunotherapy strategies

**DOI:** 10.3389/fimmu.2023.1257254

**Published:** 2023-09-01

**Authors:** Jiuhui Xu, Qianyu Shi, Boyang Wang, Tao Ji, Wei Guo, Tingting Ren, Xiaodong Tang

**Affiliations:** ^1^Department of Musculoskeletal Tumor, Peking University People’s Hospital, Beijing, China; ^2^Beijing Key Laboratory of Musculoskeletal Tumor, Peking University People’s Hospital, Beijing, China

**Keywords:** chordoma, tumor immune environment, immune-excluded, immune checkpoint inhibitor (ICI), immunotherapy

## Abstract

Chordoma is a rare malignant bone tumor with limited therapeutic options, which is resistant to conventional chemotherapy and radiotherapy, and targeted therapy is also shown with little efficacy. The long-standing delay in researching its mechanisms of occurrence and development has resulted in the dilemma of no effective treatment targets and no available drugs in clinical practice. In recent years, the role of the tumor immune microenvironment in driving tumor growth has become a hot and challenging topic in the field of cancer research. Immunotherapy has shown promising results in the treatment of various tumors. However, the study of the immune microenvironment of chordoma is still in its infancy. In this review, we aim to present a comprehensive reveal of previous exploration on the chordoma immune microenvironment and propose promising immunotherapy strategies for chordoma based on these characteristics.

## 1 Introduction

Chordoma is a rare, slow-growing, low-to-moderate malignant bone tumor that originates from residual embryonic notochord. The common sites of chordoma are axial bones, with the sacrum accounting for over 50% of primary sacral tumors (1). In adults, 50% of chordoma involve the sacrococcygeal region, 35% occur in the skull base, and 15% are found in other parts of the spine. The incidence of chordoma is low, with only 0.08 cases per 10,000 people (2), presenting a male predominance, commonly found in 50- to 60-year-old male patients; however, chordomas located in the skull base tend to occur at younger ages, even in adolescents and children. The median survival time for patients with chordoma is 6.29 years, with overall survival rates of 67.6% at 5 years and 39.9% at 10 years (3). Although chordoma are slow-growing, their robust local invasiveness leads to a high rate of local recurrence after surgery. The local recurrence rate has become the strongest predictor of mortality for patients with chordoma (4). The advanced chordoma often metastasize to multiple organs in the body, such as the lungs, bones, soft tissues, lymph nodes, and liver. Once distant metastases occur, the median survival time drops to less than 12 months (5).

Chordoma is resistant to chemotherapy (6, 7) and exhibits some degree of resistance to radiation therapy, which may be because of the low proliferation pool of cells (8), with no effective treatment for chordoma. Additionally, high doses of radiation are limited due to the low tolerance of adjacent tissues such as the spinal cord, rectum, bladder, and brainstem to the therapeutic dose for chordoma.

A wealth of evidence has emerged revealing how the functionality of the tumor immune microenvironment (TIME) determines its integral and indispensable role in tumor initiation and progress (9). Immunotherapy by targeting TIME and potentiating host anti-tumor immune responses has gradually become a hot topic in the field of cancer treatment and has been clinically applied in various types of tumors (10, 11). Immunotherapy is expected to improve the prognosis of patients with chordoma. However, there has been no review comprehensively sketching the TIME of chordoma by far; in this review, we aim to investigate the immune microenvironment studies of chordoma and propose rational and effective immunotherapeutic strategies for the patients with chordoma.

## 2 The basic research on chordoma

### 2.1 The role of brachyury in chordoma

Brachyury, a T-box family transcription factor, plays a critical role in the development of the notochord during embryogenesis and is only minimally expressed in adult tissues, such as the pituitary gland, thyroid, and testes (12). Brachyury not only is used as a diagnostic marker for chordoma, but also serves as a key driver in the development and progression of chordoma. Sharifnia et al. found that brachyury is highly enriched in multiple chordoma cell lines and mediate the overexpression of genes involved in malignant progression, such as extracellular matrix (ECM) regulation and EGFR-related signaling pathways, by binding to super enhancers in chordoma (13). Experimental evidence has demonstrated that knockdown of brachyury expression by shRNA can inhibit the progression of chordoma (14, 15). However, similar to other transcription factors, brachyury is difficult to target with drugs, and currently there are no targeted drugs or small-molecule inhibitors available for brachyury. Clinical trials of a brachyury-targeting vaccine (GI-6301) have also been declared ineffective (16).

### 2.2 Non-coding RNAs in chordoma progression

In recent years, more and more studies have shown that non-coding RNAs (ncRNAs) play an important role in the occurrence and development of various diseases by regulating gene expression (17, 18). Currently, research on ncRNAs in chordoma mainly focuses on miRNA and lncRNA (19–21). For example, Zhang et al. found that miR-16-5p can inhibit chordoma proliferation, migration, and invasion by regulating downstream Smad3 expression (21). Lou et al. further revealed that circTLK1 can regulate the biological functions of miR-16-5p through sponge adsorption, thus forming a circTLK1/miR-16-5p/Smad3 positive feedback loop to promote chordoma invasion, migration, and epithelial–mesenchymal transition (22). Revealing the competing endogenous RNA (ceRNA) network in chordoma can help to better explore the developmental mechanisms of chordoma. Furthermore, the ceRNA network also plays an important role in chemotherapy resistance, tumor prognostic markers, and other aspects of various tumors (23, 24), which require further investigation in chordoma.

### 2.3 Research on signaling pathways in chordoma

**Figure 1:**
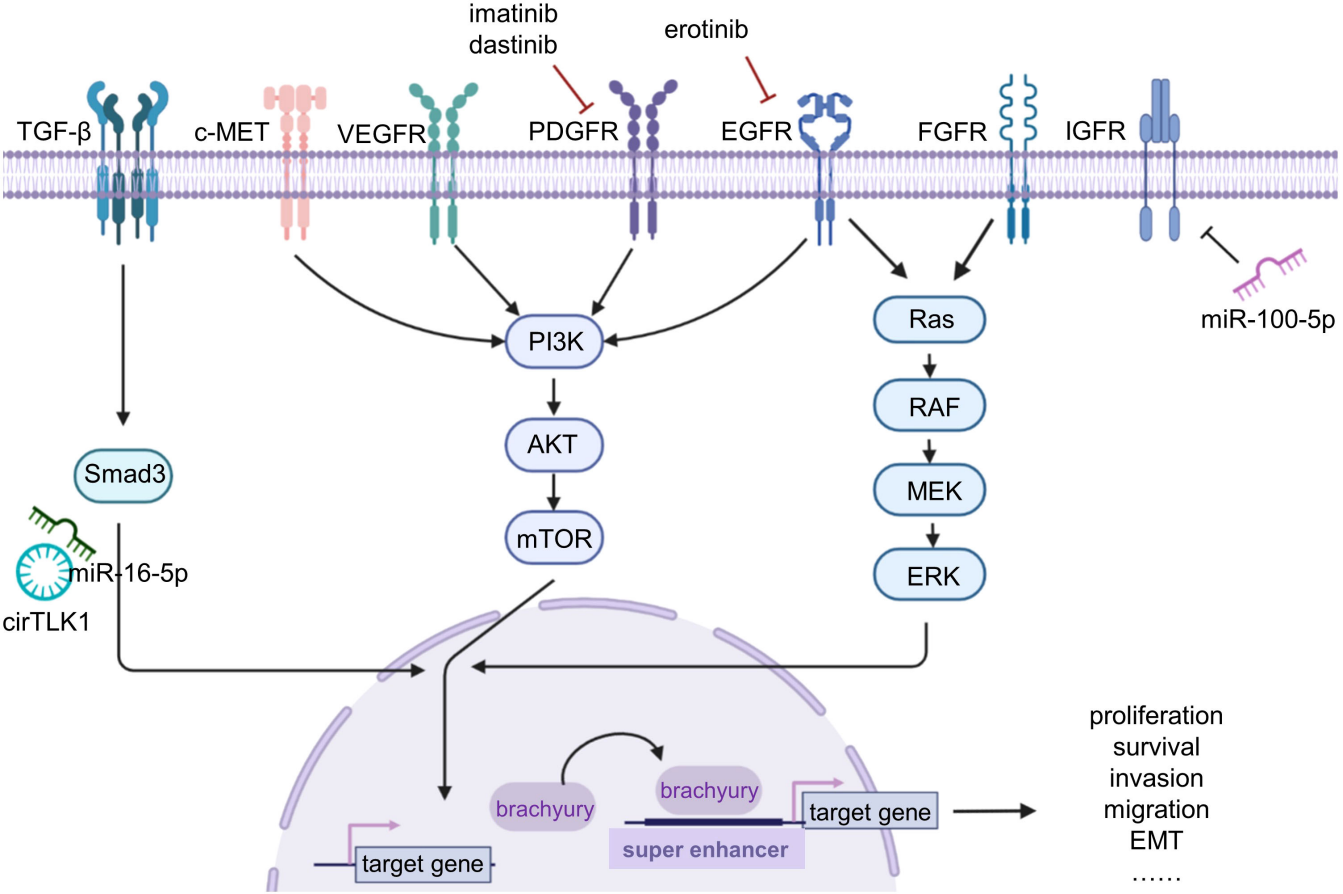
The basic research on chordoma. Chordoma studies mainly focus on the signaling pathway, brachyury, and non-coding RNAs. Various receptors and signaling pathways are dysregulated in chordoma; using imatinib, dastinib, and erotinib can inhibit the corresponding signaling pathways for targeted therapy. Brachyury plays a driving role in chordoma, causing a variety of malignant genes upregulated by binding to super enhancers. Non-coding RNAs are also dysregulated and lead to tumor progression.

Current research has shown that multiple mutations or overexpression of receptor tyrosine kinases (RTKs) occur in chordoma tissue and cell lines. Currently, the dysregulated signaling pathways in chordoma mainly focus on platelet-derived growth factor receptor (PDGFR) (25), vascular endothelial growth factor receptor (VEGFR), epidermal growth factor receptor (EGFR) (26), human epidermal growth factor receptor 2 (HER2/neu) (26), phosphatidylinositol 3-kinase (PI3K)/protein kinase B (Akt)/mammalian target of rapamycin (mTOR) (27), fibroblast growth factor (FGF)/mitogen-activated protein kinase kinase (MEK)/extracellular signal-regulated kinase (ERK) (28), hepatocyte growth factor receptor (HGFR, c-Met) (29), and insulin-like growth factor 1 receptor (IGF-1R)-related signaling pathways (30). Among these pathways, multiple pathways can mediate the expression of brachyury, which may be a key pathway driving the occurrence and development of chordoma (**Figure 1**). In addition to these signaling pathways, scattered studies have also revealed other signaling pathways involved in chordoma. For example, Shihabi et al. (31) constructed a patient-derived organoid model of chordoma and conducted high-throughput drug screening, and found that the NF-κB pathway and MUC1 were dysregulated in chordoma. Corresponding molecular targeted therapy clinical trials have been conducted for the relevant RTKs and signaling pathways in advanced and recurrent cases of chordoma.

## 3 Tumor immune microenvironment

### 3.1 Immune cells interacted with chordoma cells

Immune cells are the cellular basis for immunotherapy, and understanding the composition and functional status of immune cells in the tumor microenvironment (TME) is crucial. Various immune cells in the body can exert anti-tumor effects, among which cytotoxic T cells (CTLs, CD8^+^ T cells) are considered to be the main effector cells. CD8^+^ T cells can be activated and recruited into the TME to exert anti-tumor effects by releasing granules and perforins (32). In addition to CTLs, NK cells, macrophages, DCs, and other immune cells can also exert certain anti-tumor effects. However, there exist abnormally complex interactions between tumor cells and the immune system. Tumor cells can create an immunosuppressive microenvironment by secreting a large number of immunosuppressive cytokines (such as IL-10, TGFβ, and PGE2), recruiting immunosuppressive cells such as regulatory T cells (Tregs), activating negative regulatory pathways (CD80/CTLA4 and PD-1/PD-L1), and overexpressing immune regulatory enzymes such as indoleamine 2,3-dioxygenase (33), resulting in immune cell exhaustion or dysfunction, and affecting the normal anti-tumor function of the immune system.

T lymphocytes are the most important immune cells that exert anti-tumor effects and there are multiple cell subtypes, including CTLs, Th cells, and Tregs, all of which play important roles in the TME (34). T cells can recognize tumor cells by binding to major histocompatibility complex I/II (MHC-I/II) molecules on the surface of tumor cells via T-cell receptors (TCRs) on their own cell membrane. The human MHC molecules are also known as HLAs, where HLA-I molecules present endogenous antigens to CD8^+^ T cells, and HLA-II molecules present exogenous antigens to CD4^+^ T cells (35). Tumor antigens are presented to T cells on the surface of HLA-I molecules, recognized by TCRs, and then activate T cells to exert their effects. Patel et al. (36) performed immunohistochemistry staining for HLA-I molecules, including HLA-A, HLA-B/C, and β2-chain on 24 chordoma samples, and found that 21 out of 24 samples showed expression defects of at least one HLA-I molecule subunit. This to some extent indicates the existence of immune escape mechanisms in chordoma and alerts us to the limitations of various HLA-dependent immunomodulatory therapies. Furthermore, CTLs need to migrate into the tumor site and infiltrate into tumor tissue in order to interact with tumor cells (37). Therefore, a better understanding of the infiltration characteristics of CTLs in solid tumors is crucial for subsequent tumor immunotherapy.

Tumor-associated macrophages (TAMs) are a type of immune cells that play important roles in the TME, and can affect tumor cell proliferation, angiogenesis, and ECM formation, thus promoting or inhibiting tumor progression (38). TAMs are usually classified into two subtypes: M1 and M2 macrophages. M1 macrophages are activated by Th1 cytokine interferon-gamma (IFN-γ) or lipopolysaccharides (LPS) and have an anti-tumor effect through complement-mediated phagocytosis of tumor cells. In contrast, M2 macrophages are activated by Th2 cytokines interleukin-4/13 (IL-4/IL-13), and play roles in fibrosis, anti-inflammatory response, and tumor progression (39–41). Macrophages are highly plastic, and can polarize into M0 subtype before differentiating into M1 or M2 subtype depending on the presence of specific cytokines (42). M2 macrophages have been shown to be associated with poor prognosis in various cancers such as colorectal cancer and bladder cancer, and promote tumor progression through mechanisms such as enhancing tumor cell proliferation, invasion, and angiogenesis (43–46). Currently, there are various therapeutic strategies targeting TAMs in the TME, such as inhibiting macrophage polarization towards the M2 subtype and enhancing macrophage phagocytosis by targeting the CD47/SIRPα signaling pathway. CD47 is a protein widely expressed on the surface of many tumor cells, and studies have shown that tumor cells overexpressing CD47 can interact with SIRPα on the surface of macrophages or dendritic cells, leading to activation of downstream SHP-1 and SHP-2 phosphatases, and inhibition of macrophage phagocytosis, thereby promoting immune escape (47). High expression of CD47 has been associated with poor prognosis in acute myeloid leukemia, ovarian cancer, and glioma (48, 49). Targeting CD47 has been shown to enhance macrophage phagocytosis and inhibit tumor growth in animal models of ovarian cancer, small cell lung cancer, breast cancer, and other cancers (48, 50).

DCs are important antigen-presenting cells (APCs) that belong to the mononuclear phagocyte system (MPS) along with monocytes and macrophages (51). There are two main subtypes of DCs, plasmacytoid DCs (pDCs) and classical/traditional DCs (cDCs) (52). pDCs are capable of producing high levels of type I interferons and play important roles in regulating innate and adaptive immunity. Type I interferons are considered a double-edged sword in tumor immunity, as they can provide inflammatory signals to activate the immune system, but can also stimulate the inhibitory feedback of immune and tumor cells (53). In addition to type I interferons, pDCs secrete multiple cytokines that can stimulate the activation of DCs and macrophages, enhance the cytotoxicity of NK cells and CD8^+^ T cells, as well as promote the formation of immunosuppressive TME by recruiting Tregs and inducing the expression of immunosuppressive molecules (53, 54). cDCs can be divided into two subgroups, cDC1s and cDC2s (55). cDC1s can present MHC-I molecule antigens to CTLs, and cDCs can secrete cytokines such as CXCL9 and CXCL10 to recruit effector T cells and NK cells to the tumor site, as well as secrete cytokines such as IL-12 and CCL5 to maintain the function of effector T cells (56–58). cDC2s mainly present MHC-II molecule antigens to CD4^+^ T cells (56); when CD4^+^ T cells migrate to lymph nodes, cDC2s can activate CD4^+^ T cells (59). Therefore, the relationship between DCs and T lymphocytes is closely related in tumor immune regulation, but there is still much to be explored in terms of their communication mechanisms.

NK cells are an important subset of lymphocytes in innate immunity that exert anti-tumor effects without the need for antigen stimulation. As a cytotoxic lymphocyte, NK cells can kill tumor cells through both the release of cytotoxic granules and antibody-dependent cellular cytotoxicity (ADCC) and engagement of death ligand receptors. Therefore, NK cells can exert their effects in the early stages of tumor development. NK cells not only secrete pro-inflammatory cytokines such as IFN-γ, TNF, and granulocyte-macrophage colony-stimulating factor (GM-CSF), but also secrete immunosuppressive factors such as TGF-β and IL-4 (60). Currently, some therapies targeting NK cells are in the exploration phase, such as transferring NK cells into tumor patients, CAR-NK cell therapy, and cytokine therapy.

### 3.2 Tumor immune microenvironment classification

Based on the infiltration of CD3^+^ and CD8^+^ T cells in the tumor center and invasive margin of the TME, the immune score system has been introduced to classify tumors into “cold” and “hot” types (61). Hot tumors are characterized by a large amount of infiltrating T cells that are activated, while cold tumors mainly exhibit a lack of T-cell infiltration (62, 63). Currently, the immune score system has been widely applied in various types of cancer, and its predictive accuracy for patient prognosis in colorectal cancer has surpassed that of the traditional TNM staging system (64). Therefore, the evaluation of tumor prognosis has shifted from a tumor-centric system to an immune-centric system. The widespread infiltration of CD3^+^ and CD8^+^ T cells is considered a hallmark of immune recognition initiated, but the immune microenvironment of tumors is complex. With the development of various research techniques, further exploration of the number, type, spatial distribution, and activation state of immune cells in the microenvironment has revealed significant heterogeneity in the composition and phenotype of tumor-infiltrating lymphocytes (TILs) (65–67). Some studies have identified the existence of CD8^+^FOXP3^+^ regulatory T cells’ subtype using single-cell sequencing and multiple immunofluorescence (mIF) techniques (68, 69). Although CD8^+^ T cells generally exhibit cytotoxicity, CD8^+^FOXP3^+^ Treg cells exhibit immunosuppressive characteristics in certain diseases. However, their function in tumor immunity still requires further exploration, and therefore, relying solely on the infiltration of CD3^+^ and CD8^+^ T cells to evaluate the immune status of tumors is incomplete.

Therefore, more new and detailed classifications of the TIME have emerged, and exploring the TIME characteristics of different tumor types can provide more personalized and rational treatment strategies (70). Through the comprehensive analysis of the immune microenvironment characteristics of multiple tumors, Galon et al. proposed a new immunological classification, which mainly divides the TIME into the following four types: immune-infiltrated/hot tumors, immune-excluded tumors, immune-suppressed tumors, and immune-desert/cold tumors (70, 71).

The immune-excluded phenotype of tumors is characterized by a typical spatial distribution pattern, where a large number of T cells are confined to the periphery of the tumor and unable to infiltrate into the tumor parenchyma or interact with tumor cells to exert their anti-tumor effects. The immune-excluded TME is composed of various mechanisms, such as vascular structure and fibroblasts that can form a physical barrier to impede T-cell infiltration (72). Additionally, there are numerous soluble molecules in the microenvironment that can suppress T-cell chemotaxis into the tumor parenchyma (73), and when T cells come into contact with tumor cells, it can trigger immune-inhibitory signaling pathways such as PD-1/PD-L1, creating a dynamic exclusion mechanism. Furthermore, some researchers have proposed that hypoxia may be a triggering factor that induces the above mechanisms in tumors (74), resulting in the immune-excluded phenotype through remodeling of the ECM, modulation of TME metabolism, and upregulation of immune checkpoint such as CD47/SIRPα (75–78).

The difference between the immune-suppressed and immune-excluded phenotypes lies in that a large number of immune cells are not restricted to the stromal regions surrounding the tumor, but rather their infiltration and expansion are limited by the immunosuppressive microenvironment (71), resulting in less immune cell infiltration in the tumor parenchyma. For example, research has revealed that low expression of IL-15 in colorectal cancer can inhibit the proliferation of B cells and T cells and increase the risk of tumor recurrence (79).

### 3.3 The exploration for chordoma TIME

Chordoma is a rare malignant bone tumor and there is limited basic research on its immune aspects. Literature on this subject only began to emerge in 2015 (80, 81), when Mathios examined PD-L1 expression in 10 samples of chordoma. This study opened the door to exploring the immune microenvironment of chordoma and revealed that PD-L1 was negative in chordoma cells, but was expressed in macrophages and T cells (80). Subsequent studies expanded the sample to 78 tissue specimens and further investigated PD-L1 expression, as well as its relationship with clinical characteristics of patients. Unlikethe findings of Mathios, these studies showed that PD-L1 was positively expressed in tumor cells and was associated with poor prognosis of patients with chordoma (81, 82). However, the infiltration of immune cells was subjectively judged only based on the histological characteristics of the cells at the time, and analyzing the infiltration of individual immune cells or the expression of PD-L1 in tumor tissue alone could not fully elucidate the TIME characteristics of chordoma. In 2016, Inagaki used immunohistochemistry to explore the immune infiltration characteristics of various bone tumors, including two cases of chordoma. The study analyzed the infiltration of various immune cells and revealed that macrophages were the major cell subtype, followed by T cells and dendritic cells (DCs), in multiple bone tumors (83).

Different subgroups of immune cells often play different roles, as M1 and M2 macrophages even have opposite functions in many diseases. Therefore, analysis of cell types needs to delve into various subtypes. Zou analyzed two subtypes of T cells (CD8^+^ TILs and Foxp3^+^ TILs) in chordoma in 2018 and found that the ratio of CD8^+^ TILs to Foxp3^+^ TILs was 3.3 times and related to the patient’s overall survival (OS) (84). This team then developed an immune scoring system based on the distribution of CD3^+^ and CD8^+^ TILs in the tumor and invasive edge in the following year, which could serve as a prognostic factor for the patient’s local recurrence-free survival (LRFS) and OS (85). In addition, the team used mIF to analyze the expression of PD-1, CD3, CD8, CD20, and FOXP3 in 2020, and validated the results by flow cytometry (86). They constructed an immune scoring system based on the density of interstitial CD8^+^ TILs, interstitial Foxp3^+^ TILs, tumor area Foxp3^+^ TILs, and tumor area PD-1^+^ TILs, which showed correlations with patient LRFS and OS. This is the first time that an objective experimental technique, different from immunohistochemistry and H&E staining, which require subjective interpretation, has been used in exploring the immune microenvironment of chordoma.

In a large-scale sarcoma study conducted in 2020 (1,242 cases, including over 27 types of sarcoma and 28 cases of chordoma), M1 and M2 subtypes of macrophages were analyzed for the first time. The results showed that the number of macrophages infiltrating the tumors was higher than that of lymphocytes in almost all sarcomas, and the infiltration of M2 macrophages was dominant. Among them, chordoma had the highest macrophage/TIL ratio among the various types of sarcoma, exceeding 10 times, and the M2/M1 ratio was 1.5 times. The expression of the macrophage-related immune checkpoint CD47/SIRP was also extremely high in chordoma, with CD47 expressed on all tumor cells in 82% of cases, and SIRP^+^ macrophage infiltration present in 71% of cases (87).

In addition to the PD-1/PD-L1 pathway of T-cell immune checkpoints, other immune checkpoints have also been studied, which can induce T-cell apoptosis by binding to their corresponding ligands. In 2019, Zhou (88) revealed that TIM3^+^ TIL infiltration is associated with invasion of chordoma. In 2020, He (89) analyzed 32 specimens of chordoma and found that CTLA-4 showed positive expression on the surface of tumor cells and TILs in all cases.

Both the immune surveillance function of the body to clear foreign invaders and T-cell therapy using immune checkpoint inhibitors (ICIs) rely on the T cells’ ability to exert cytotoxic effects. The premise for T cells to exert their effector function is to recognize the human leukocyte antigen-I (HLA-I) molecules on the surface of tumor cells and the tumor antigens. However, studies have found that HLA-I expression is downregulated in 21 out of 24 cases of chordoma (36), indicating that the cytotoxic ability of T cells may be limited in chordoma, and immunotherapy relying on T cells may have limited efficacy.

### 3.4 Overall immune landscape of chordoma

**Figure 2:**
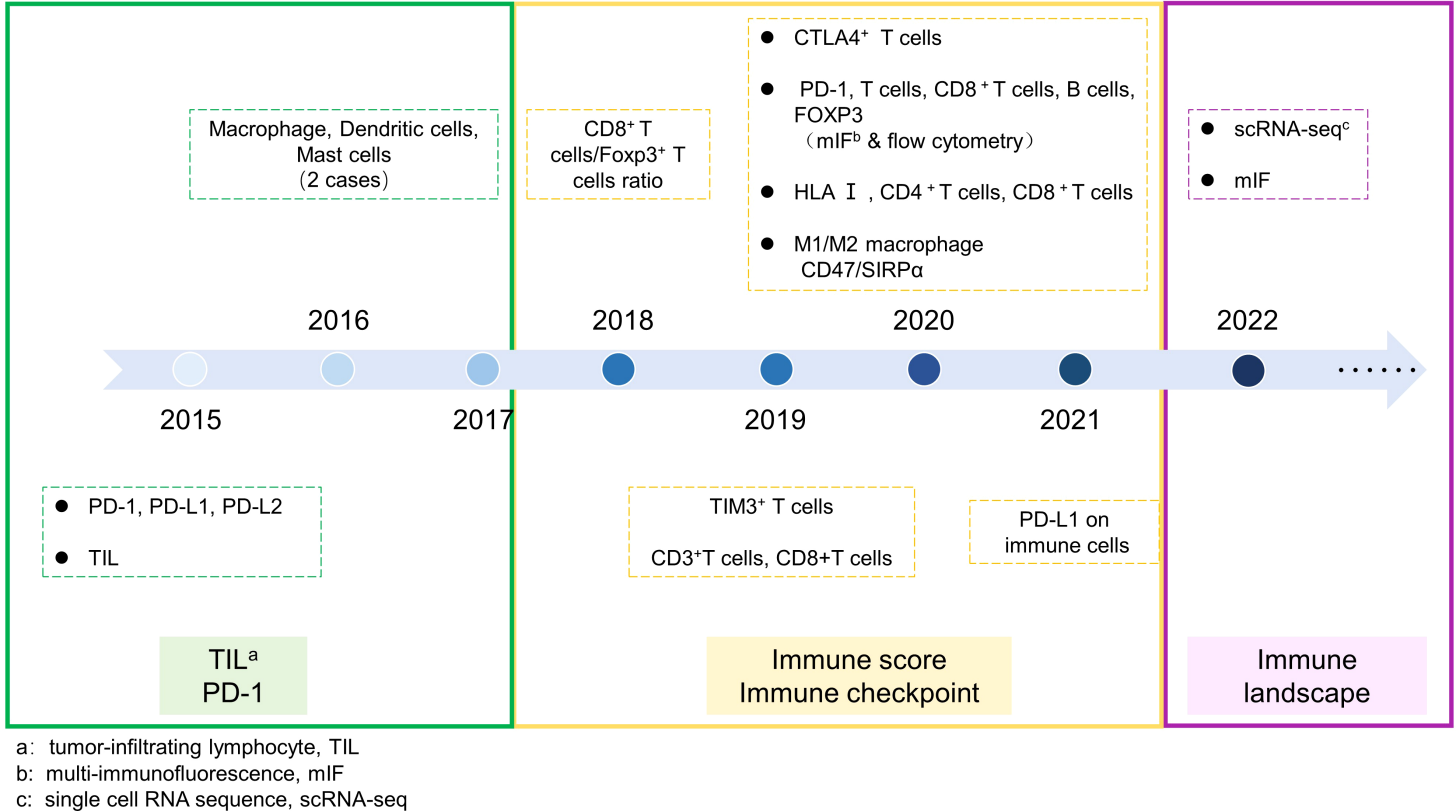
Exploration of chordoma immmune microenvironment. The timeline of chordoma immune microenvironment expolration was divided into three periods. Period 1: Focuses on the expression of PD-1 signaling pathway and infiltration of individual TIL. Period 2: Exploration of various immune checkpoints and comparison of infiltration of multiple immune cells. Period 3: High-dimensional assessment of the overall immune landscape of chordoma.

In recent years, single-cell sequencing technology (scRNA-seq) has made great strides. Unlike traditional transcriptome sequencing technologies, scRNA-seq can comprehensively reveal gene expression at the single-cell level. Currently, it has been widely applied in various research studies. In 2022, more objective and comprehensive exploration of chordoma was conducted through single-cell sequencing and mIF (90, 91) (**Figure 2**). Duan et al. first used scRNA-seq to depict the gene expression profile of chordoma and analyze tumor cells, immune cells, and fibroblasts in the microenvironment (91). This marks the beginning of a high-throughput era for exploring the immune microenvironment of chordoma. Duan found that mononuclear phagocytes were the most prominent infiltrating immune cell (14.0%). Based on the gene expression characteristics of macrophages, M2 macrophages were widely infiltrated and may play a critical role in chordoma angiogenesis. T cells and NK cells (11.7%) only expressed a small amount of immune checkpoint, suggesting that various ICIs targeting T and NK cells may have poor efficacy in chordoma.

Owing to the rarity of chordoma, there is currently a limited number of studies on its immunemicroenvironment and the number of samples involved is relatively small. In some cases, the conclusions may even be conflicting. Therefore, we are still unable to make further judgments on the overall status of the immune microenvironment of chordoma. Further research is needed to provide a more comprehensive and in-depth description of the immune microenvironment of chordoma.

## 4 Immunotherapy strategies for chordoma

### 4.1 Immune checkpoint inhibitor therapy

Immune checkpoint pathways are inhibitory signaling pathways that exist within the immune system. ICI therapy works by blocking the immune checkpoint pathway, and releasing the “brakes” on immune cells to restore their anti-tumor function. Under normal physiological conditions, immune checkpoints are designed to prevent overactivation of immune cells. The emergence of tumors is a result of mutations in normal cells. Immune checkpoint on the surface of immune cells can bind to corresponding receptors on the surface of tumor cells and cause immune dysfunction through various pathways, such as inhibition of T-cell proliferation, survival, cytokine secretion, and other effector functions, leading to immune escape.

Currently, the most extensively studied checkpoint pathways in the field of cancer research are programmed death protein 1 (PD-1/CD279) and its ligands PD-L1/CD274/B7-H1 and PD-L2/CD273/B7-DC, cytotoxic T lymphocyte-associated antigen 4 (CTLA-4), T-cell immunoglobulin and mucin domain 3 (TIM3), and lymphocyte activation gene 3 (LAG3), among others (92).

PD-1 and PD-L1 both belong to the immunoglobulin superfamily type I transmembrane proteins. PD-1 is expressed on the surface of various immune cells such as T cells, B cells, NK cells, and DC cells (93), while PD-L1 is mainly expressed on the surface of tumor cells and tumor-infiltrating lymphocytes. The binding of PD-L1 on tumor cells with PD-1 on T cells can cause a conformational change in PD-1, leading to the phosphorylation of the cytoplasmic immune receptor tyrosine-based inhibitory motif (ITIM) and immune receptor tyrosine-based switch motif (ITSM). The phosphorylated tyrosine-based motifs subsequently recruit SHP-2 and SHP-1 proteins, which weaken T-cell activation signals and inhibit T-cell activation. In addition, many studies have focused on the expression regulation mechanisms of PD-L1 at multiple levels. For example, at the gene level, Roemer et al. (94) found that 97% of classical Hodgkin lymphoma cases had mutations in the PD-L1/PD-L2 gene locus. At the protein level, Burr et al. (95) found that the protein CMTM6 can maintain the stable expression of PD-L1 on the cell membrane by binding to it, and participate in inhibiting T-cell activation and promoting tumor progression.

Currently, studies revealed that PD-L1 expression in chordoma is associated with tumor metastasis and tumor staging, and is a poor prognostic factor for both LRFS and OS. However, there is no consistent opinion among studies when analyzing the expression of PD-L1 on tumor cells and immune cells. This may be due to the inadequate sample used in the studies, but to some extent, it shows the potential for targeted PD-1/PD-L1 therapy. The expression and regulatory mechanisms of immune checkpoints on the surface of chordoma still need to be explored in depth. Currently, several drugs targeting the PD-1/PD-L1 pathway in chordoma are undergoing clinical trials (96, 97) (**Table 1**). Pembrolizumab and nivolumab are monoclonal antibodies targeting PD-1 and have demonstrated robust efficacy in areas such as melanoma and non-small cell lung cancer. Pembrolizumab has become the most widely used drug in ICI therapy for chordoma (98). It has been shown that DCs and macrophages in the chordoma microenvironment highly express regulators of PD-L1, such as AXL, TLR3, and CD40, suggesting that our treatment targeting the PD-1/PD-L1 axis in combination with blockade of the above molecules may provide better therapeutic efficacy (99).

**Table 1:** Immunotherapy clinical trial for chordoma.

Year	Immunotherapy strategy	Registration number	Sample of chordoma	Sample of all patients	Study phase	Drug
2023	ICI	NCT03012620	34	97	Phase 2	Pembrolizumab
2022	ICI	NCT02815995	5	57	Phase 2	Durvalumab (anti-PD-L1) + tremelimumab (anti-CTLA-4)
2021	Tumor vaccine	NCT04134312	10	13	Phase 1	MVA-BN-brachyury-TRICOM
2021	Tumor vaccine + radiotherapy	NCT02383498	24	24	Phase 2	GI-6301 + radiotherapy
2020	Tumor vaccine	NCT03349983	3	10	Phase 1	BN-brachyury (MVA‐brachyury + FPV-brachyury)
2017	ICI	NCT01772004	1	53	Phase 1	Avelumab (anti-PD-L1)
2017	Tumor vaccine	NCT02179515	13	38	Phase 1	MVA-brachyury-TRICOM
2015	Tumor vaccine	NCT01519817	11	34	Phase 1	GI-6301

In addition, studies have shown that the expression levels of CTLA-4 and TIM3 are both high in chordoma, and their expression is related to the invasiveness and recurrence of chordoma (88). In addition to immune checkpoints on T cells, the role of immune checkpoints on other immune cells is gradually being revealed. TAMs infiltrate heavily in chordoma and are mainly M2 macrophages, which participate in the formation of the immunosuppressive microenvironment of chordoma. CD47, SIRPα, and TIM3 are all expressed on TAMs, and chordoma CD47 expression shows the highest degree of expression among various sarcomas (87, 91), However, whether it can be used as a prognostic factor and therapeutic target for chordoma is currently unknown.

To date, the ICI therapy for chordoma is mainly limited to targeting PD-1/PD-L1, while research on other target points is relatively scarce and only limited to preclinical stages, which seriously hinders the selection of ICI therapy strategies for chordoma. Moreover, the current ICI therapy strategy only benefits a subset of patients and most patients do not show sustained remission. In addition, immune-related adverse events (IRAEs) such as myocarditis and colitis have been observed in some patients (100). This may be due to the fact that anti-tumor immune regulation requires the complex TME to exert its effects (101), and there are heterogeneities in the expression of immune checkpoints, differences in the distribution of immune cells, and interference from other inhibitory pathways in different individuals. Therefore, it is urgent to better understand the status and regulatory mechanisms of the chordoma microenvironment to guide the safer and more effective use of ICI therapy strategies (102, 103).

### 4.2 Adoptive cell therapy

Adoptive cell therapy (ACT) is one type of cellular immunotherapy, and it involves the transfer of immune cells infused into patients where they can mediate strong anti-tumor responses, resulting in tumor regression (104). Because of the HLA expression defects in chordoma cells, some T cell-dependent immunotherapy strategies may be limited. ACT involves the infusion of modified immune cells into patients, and unlike T cells that rely on the combination of TCR and tumor cell surface HLA molecules, ACT can specifically recognize the tumor cell surface antigen to exert immune effects and attack tumor cells to achieve anti-tumor goals. This may provide an effective treatment strategy for chordoma patients with downregulated HLA molecules. Among them, chimeric antigen receptor T-cell therapy (CAR-T) is currently a hot spot in immunotherapy due to its strong specificity and multiple advantages.

CAR is a cell surface receptor synthesized artificially *in vitro*, mainly composed of a single-chain variable fragment (scFv) of monoclonal antibodies in the extracellular domain, a CD3ζ molecule in the intracellular domain, and a transmembrane domain that couples them together. The extracellular domain of scFv does not rely on HLA molecules on the surface of tumor cells to present antigens, but specifically recognizes the surface antigen of tumor cells, thereby activating the signal structure domain of the intracellular domain.

By isolating T cells from patients and genetically modifying them using virus vectors and other methods *in vitro*, T cells can stably express CARs with specificity, which are then expanded and infused into patients. With the advancement of technology and research, second-, third-, and fourth-generation CARs have emerged. Second-generation CARs enhance the effect of CAR-T cells by adding a co-stimulatory structure such as CD28, CD137/4-1BB to the intracellular domain. Third-generation CARs contain two co-stimulatory domains linked to CD3ζ, which can enhance and prolong T-cell activation time (105). Fourth-generation CAR-T cells, also known as TRUCK, not only increase the co-stimulatory structure domain but can also locally secrete cytokines such as IL-12 after targeting tumor cells, enhance T-cell activation, recruit immune cells to attack other tumor cells not recognized by CAR-T cells, and regulate vascular generation to improve the immunosuppressive microenvironment of solid tumors (106).

The CAR-T cells recognize tumor antigens similar to antigen–antibody reactions and have stronger specificity compared to other immunotherapies. Currently, CAR-T cell therapy has shown good efficacy in the treatment of hematologic malignancies, and several CD19-targeted CAR-T cell products such as Kymriah and Yescarta have been approved by the FDA for the treatment of B-cell acute lymphoblastic leukemia and diffuse large B-cell lymphoma (107, 108). However, the efficacy of CAR-T therapy in solid tumors is not as significant as in hematologic malignancies, mainly due to the immunosuppressive microenvironment of solid tumors, which limits the migration of CAR-T cells to tumor sites. Even if they migrate to the tumor site, the surrounding immunosuppressive cells and cytokines can also affect the further effectiveness of CAR-T cells (109). Therefore, current studies not only focus on the structure of CAR on immune cells but also attempt to explore how to modify immune cells to enhance immunotherapy and reduce toxicity. For example, by increasing the receptor for chemokine on CAR-T cells, immune cells can infiltrate into the tumor stroma more rapidly (110, 111), knocking out the inhibitory receptors on the cell surface to inhibit early immune cell exhaustion (112), and embedding dual CAR targeting two antigens on the same immune cell to avoid the occurrence of immune escape caused by the loss of a single antigen (113).

However, CAR-T therapy has shown slow progress in solid tumors, especially in rare tumors like chordoma. Currently, there is only one article on CAR-T cell therapy for chordoma, in which Long et al. (114) constructed CAR-T cells targeting B7-H3 and demonstrated anti-tumor efficacy *in vitro*. However, the positivity rate of B7-H3 expression in chordoma is only 16%, suggesting that CAR-T cells targeting B7-H3 may only have anti-tumor efficacy in a few patients. CAR-T therapy relies primarily on identifying special targets, and ideal tumor antigens should have high specificity and only be expressed in tumor cells. However, little is known about specific antigens for chordoma at present.

### 4.3 Tumor vaccine therapy

A tumor vaccine therapy involves administering low doses of inactivated antigens to activate the immune response in the body of patients (115). Brachyury is a transcription factor found in multiple tumors that can promote EMT. It is expressed in almost all patients with chordoma, and chordoma-targeted tumor vaccines are developed based on this, including the Yeast-Brachyury (GI-6301) vaccine (16, 116), the MVA-Brachyury-TRICOM vaccine constructed using the modified vaccinia Ankara (MVA) carrier, and subsequent modified vaccines (117–119). Clinical benefits have been demonstrated in chordoma patients receiving tumor vaccines, and trials have found that pre-treatment with ICI or radiotherapy can enhance the therapeutic efficacy of vaccines, providing a potential new treatment approach for chordoma patients. However, unfortunately, a phase II trial of radiotherapy combined with the Yeast-Brachyury vaccine (GI-6301) in patients with advanced unresectable chordoma showed no complete response (CR) and the efficacy was not satisfactory, leading to termination of the clinical trial before reaching the endpoint (16) (**Table 1**).

### 4.4 Breaking the immunosuppressive microenvironment

**Figure 3:**
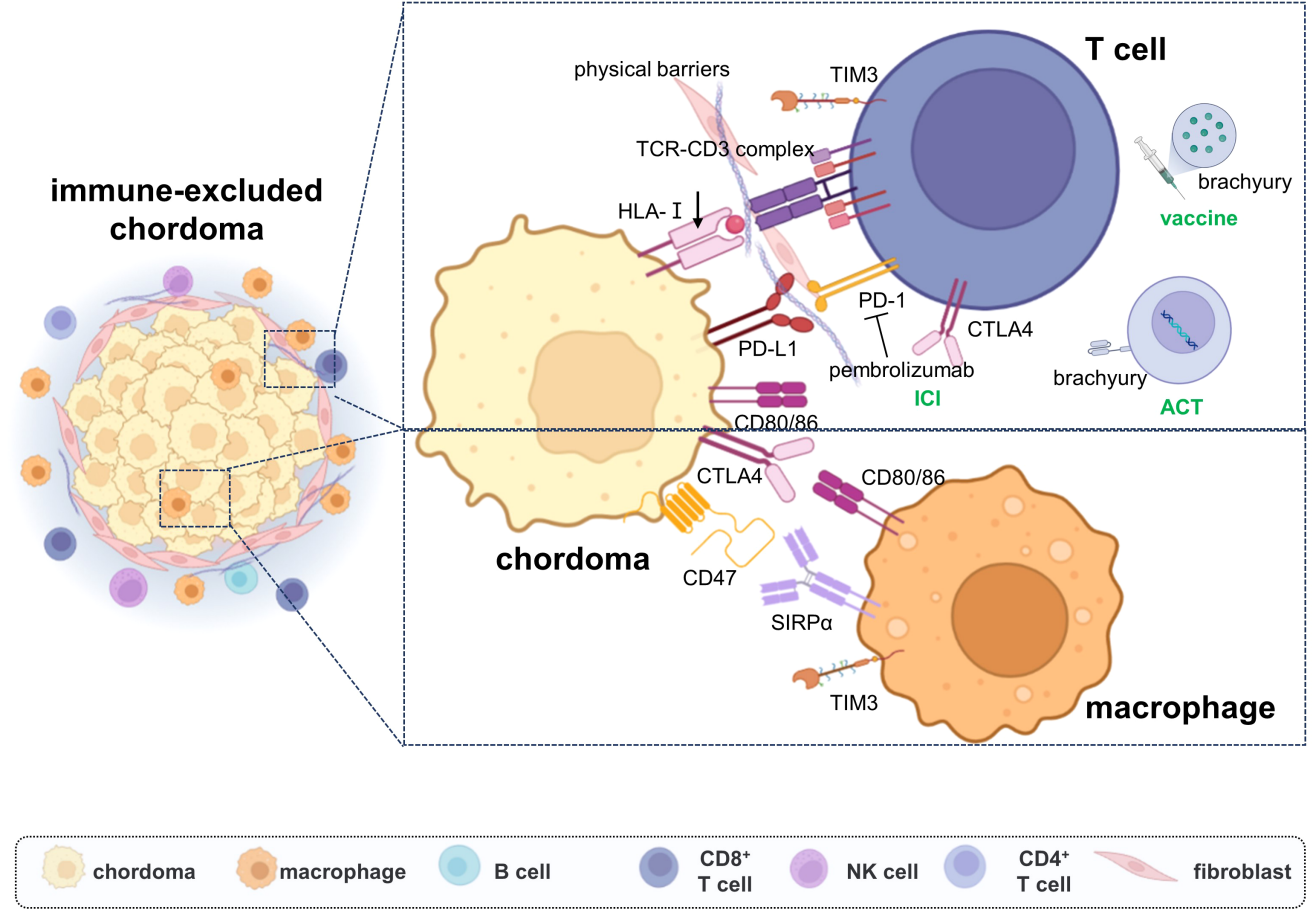
The immune escape mechanisms of chordoma and immunotherapy strategies. Chordoma was shown with immune-excluded microenvironment, the fibroblasts impede the infiltration of immune cells. Multiple immune checkpoints were upregulated on the tumor cells’ and TIL cells’ surfaces, which inhibit the immune response effect. The current immunotherapy strategies are mainly focused on immune checkpoint inhibitor therapy, with ACT and tumor vaccine also emerging.

Overall, “hot tumors” often exhibit better responses to immunotherapy, so many studies are trying to convert “cold tumors” into “hot tumors” to enhance their therapeutic effects, which is a new direction of current research. According to existing data, although we do not have a clear understanding of the specific cell phenotypes and infiltration proportions in the microenvironment of chordoma, numerous studies have shown that immune cells, especially T cells, mainly infiltrate the fibrous septa in the stroma of chordoma, exhibiting immune-excluded phenotypes (**Figure 3**). Therefore, breaking their immune suppression from multiple directions based on their spatial distribution characteristics may open up new therapeutic strategies for chordoma.

Cancer-associated fibroblasts (CAFs) are the main cell component of tumor stroma and are currently a hot target for tumor therapy, and research has shown that activation of the TGF-β pathway may be the molecular mechanism underlying the immune exclusion phenotype. TGF-β is a typical immunosuppressive molecule that can act on surface receptors of fibroblasts, indirectly affecting the localization and migration of immune cells in tumors by increasing the expression of immunosuppressive cytokines and regulating various ECM components such as collagen, α-SMA, and versican, thereby forming an immune-excluded phenotype that affects the occurrence, development, and metastasis of tumors (120). It has been revealed that both TGF-β and its multiple receptors are highly expressed in chordoma, where TGF-β is mainly secreted by microenvironmental fibroblasts and macrophages, which can both drive tumor cell EMT progression and enhance their own malignant ability, and also regulate the immune microenvironment by converting CD4^+^ T cells into Treg cells, regulating NK cells and other functions.

Inhibiting CAF activation includes the use of inhibitors of focal adhesion kinase (FAK), fibroblast growth factor receptor (FGFR), and TGF-β (121–123), as well as downstream pathways that regulate the TME by inhibiting the activation of fibroblasts, including the use of angiotensin receptor blockers and degradation of hyaluronic acid produced by fibroblasts (124, 125), which are all expected to have a regulatory effect on the TME. Tumor progression in chordoma has been found to be inhibited by blocking the TGF-β pathway (126), but the regulatory effects on the fibroblast in TME remain to be further explored.

## 5 Conclusion

As a rare malignant bone tumor, the research of chordoma has been slow. Macrophages and T cells were dominated in the chordoma microenvironment, which are likely to be the crucial effector cells in tumor immune regulation and may be the therapeutic targets for chordoma immunotherapy. HLA-I is downregulated in chordoma, suggesting that T cell-dependent immunotherapy strategy may be impeded. Various classic immune checkpoints and immune-excluded phenotype were progressively revealed, which may be the essential mechanisms of chordoma immune escape.

## 6 Future directions

The limited basic research of chordoma has severely restricted the choice of clinical treatment strategies. Previous studies have focused mainly on the driving mechanisms of tumor cells themselves, but tumor progression is not limited to the tumor cells alone. The exploration of the immune microenvironment of chordoma started in 2015, and by summarizing the findings so far, we found that macrophages and T cells were the most highly infiltrated in chordoma and may be the crucial effector cells in tumor immune regulation. A variety of immunosuppressive checkpoints such as PD-1/PD-L1, CD47/SIRPα, TIM3, and CTLA4 are expressed on the surface of both chordoma cells and immune cells, and ICIs are the most widely performed strategy in the field of chordoma immunotherapy. It is worthwhile to further investigate the molecular regulation mechanisms of immune checkpoints and whether the dual-target immunotherapy can achieve better efficacy.

However, HLA-I is downregulated on the surface of chordoma cells, which is a prerequisite for T cells to exert tumor-killing effects, suggesting that T cell-dependent immunotherapy strategy may be impeded. Deciphering the intricate mechanism of HLA downregulation is a direction worthy of further investigation, and whether CAR-T therapy, which does not depend on the identification of TCR-HLA, can be a promising approach to immune-mediated chordoma treatment. Similar to tumor vaccines, the efficacy is highly dependent on the specificity of the tumor antigens; therefore, specific chordoma tumor neoantigens need to be immediately identified.

At the same time, we found that chordoma exhibits an immune-excluded phenotype, with a large number of immune cells infiltrating the mesenchymal region and preventing it from exerting an immune response. Exploring the genetic differences between parenchymal immune cells and mesenchymal immune cells may provide novel therapeutic targets for chordoma and alter its immune-excluded phenotype.

Brachyury and various signaling pathways are revealed to be associated with the malignant capacity of chordoma cells, but their functions on chordoma immune microenvironment remains to be unclear. For example, the VEGF and TGF-β pathways are dysregulated in chordoma, whether inhibiting the VEGF pathway could modulate the vascular structure of the microenvironment and whether targeting TGF-β could inhibit its effect on fibroblasts, which creates a tough physical barrier. Thus, altering the immune-excluded phenotype will hopefully improve the efficacy of various immunotherapeutic strategies.

## Author contributions

JX: Data curation, Writing – original draft, Writing – review & editing. QS: Data curation, Writing – original draft, Writing – review & editing. BW: Data curation, Writing – original draft. TJ: Supervision, Writing – original draft. WG: Supervision, Writing – review & editing. TR: Funding acquisition, Supervision, Writing – review & editing. XT: Funding acquisition, Supervision, Writing – review & editing.
